# Adult Cystic Lymphangioma of the Parotid Gland: An Unwonted Presentation

**DOI:** 10.7759/cureus.2644

**Published:** 2018-05-18

**Authors:** Sakthivel Chinnakkulam Kandhasamy, Thangadurai Ramasamy Raju, Ashok Kumar Sahoo, Gopalakrishnan Gunasekaran

**Affiliations:** 1 General Surgery, Jawaharlal Institute of Postgraduate Medical Education and Research (JIPMER), Puducherry, IND; 2 Department of General Surgery, Vardhman Mahavir Medical College and Safdarjung Hospital, New Delhi, IND

**Keywords:** ultrasonography, magnetic resonance imaging, fine needle aspiration cytology, cystic lesion

## Abstract

Cystic lymphangioma of the parotid gland is an uncommon congenital lymphatic malformation. Its occurrence in patients of advanced age is infrequent. Patients usually present with painless soft swelling, often having experienced a long duration of symptoms. Lymphangioma among the salivary glands frequently involves the parotid gland. When evaluating cystic lesions of the parotid gland, cystic lymphangioma should be included in the differential diagnosis in addition to Warthin’s tumor, branchial cyst, cystic pleomorphic adenoma, and cystic mucoepidermoid tumor. Ultrasonography (USG) and magnetic resonance imaging (MRI) are useful in diagnosing cystic lymphangioma and help to identify the lesion. Fine needle aspiration cytology (FNAC) may show lymphocytes, salivary epithelial cells, and rarely, endothelial cells. FNAC is often inconclusive; this was the case in our investigation of the cystic lesion presented here of a 50-year-old woman who presented with a slowly growing swelling and a dull aching pain over the right parotid region for the past two years. On examination, there was a non-tender, cystic swelling of 5×5 cm in the right parotid region causing lifting the earlobe. There was no cervical lymphadenopathy or any facial nerve palsy associated with the swelling. USG of the parotid gland revealed a cystic lesion in the superficial lobe of the parotid. Results of FNAC performed on the lesion were inconclusive. The patient was posted for surgery and the cyst was excised. Final histopathology of the lesion gave the diagnosis of cystic lymphangioma of the parotid gland. The patient was kept under follow up for six months to watch for any local recurrence, but none occurred.

## Introduction

Cystic lymphangioma is a benign lesion consisting of multiple lymphatic spaces. Lymphangiomas are benign lymphatic malformations most frequently observed in the pediatric age group and, uncommonly, in the elderly population [1]. The head and neck are the most common locations for lymphangioma to occur. Other less common sites are the axilla, shoulder, chest and abdominal wall [2]. Parotid gland lymphangioma is a rare occurrence in adults. To date, there are only very few cases of adult cystic lymphangioma of the parotid. Preoperative diagnosis is difficult in adults and often patients are misdiagnosed. Definitive diagnosis can be reached only after histopathological examination of the surgical specimen. Here, we present the case of a 50-year-old woman with cystic lymphangioma of the right parotid gland which is uncommon.

## Case presentation

A 50-year-old post-menopausal female presented to the outpatient surgical department with complaints of a slowly growing swelling and a dull aching pain over the right parotid region for the past two years. She had no past history of trauma, surgery or infection of the right parotid. On examination, there was a non-tender, cystic swelling of 5×5 cm in the right parotid region causing lifting of the earlobe. The swelling became more prominent on clinching the teeth. The swelling was not fixed to the skin or any underlying structures. Examination of the oropharyngeal and facial nerves showed no abnormalities. There was no cervical lymphadenopathy.

Routine blood tests were within normal limits. Ultrasonography (USG) of the parotid gland revealed a cystic lesion measuring 5×6 cm involving the superficial lobe of the right parotid gland. Fine needle aspiration cytology (FNAC) performed on the lesion was inconclusive.

**Figure 1:**
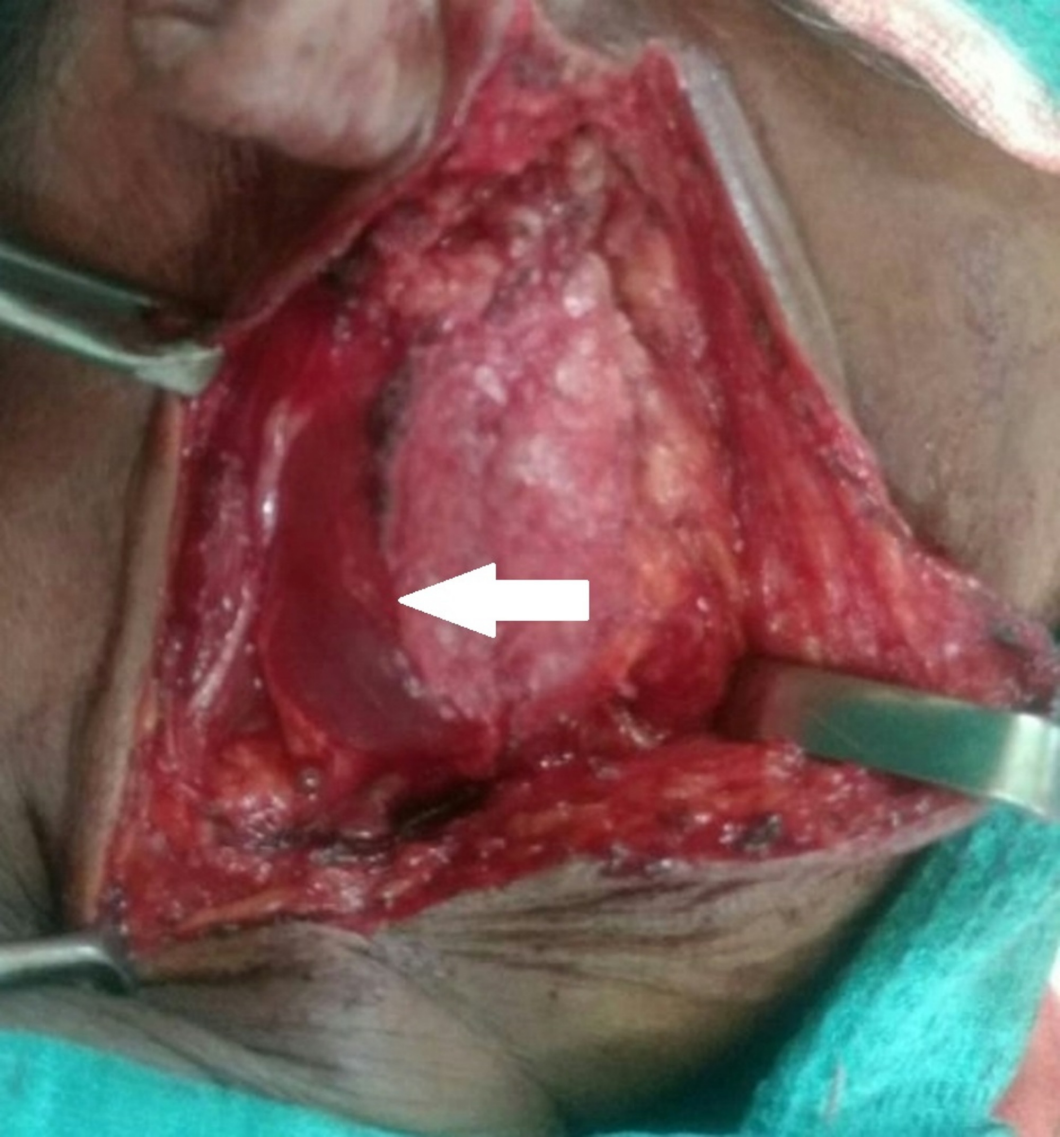
Multilocualated cyst arising from the superficial lobe of the parotid gland (arrow)

The patient was posted for surgery. Intra-operatively, it was discovered that she had a multiloculated cyst arising from the superficial lobe of the right parotid (Figure 1).

**Figure 2:**
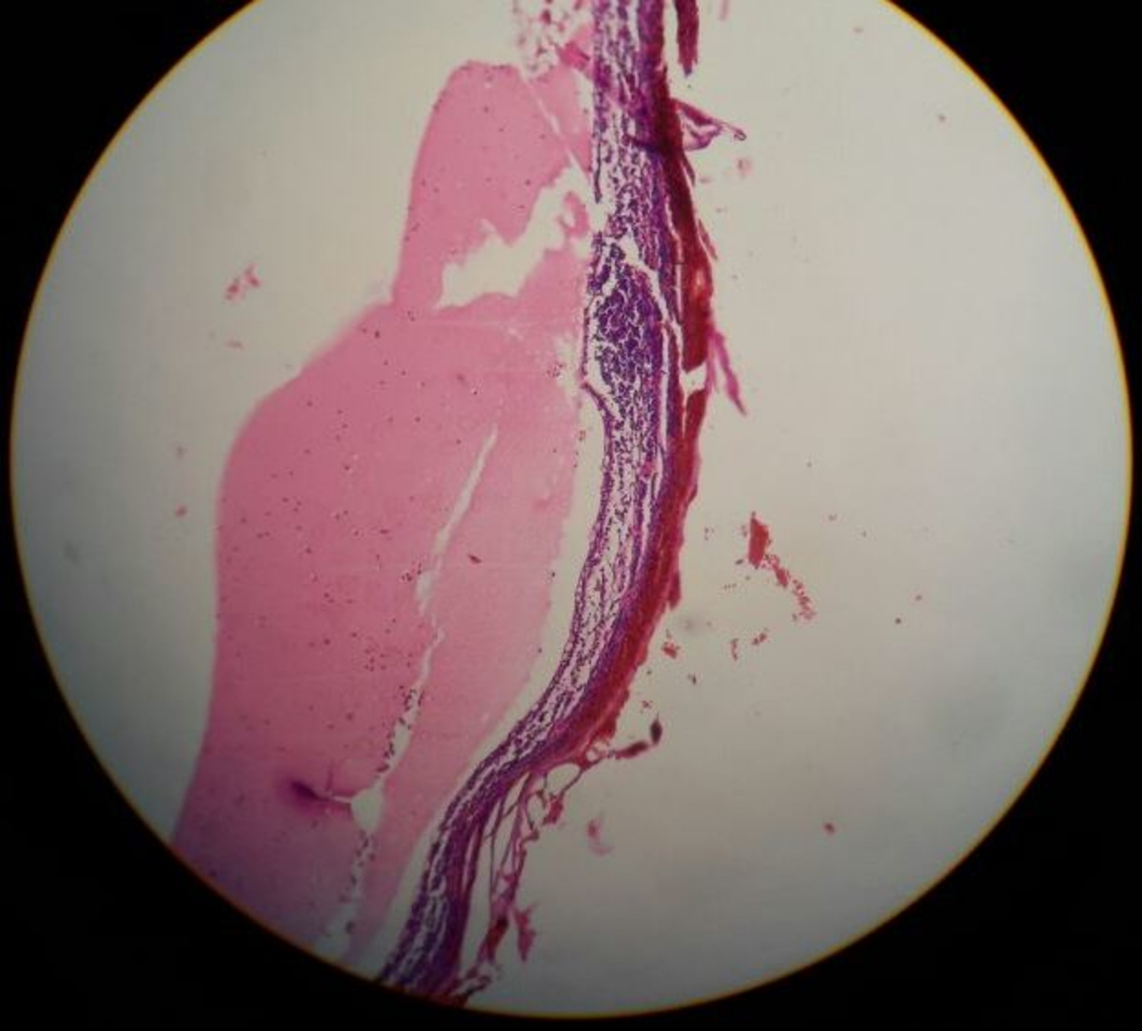
Pathological section showing the cyst wall consisting of fibrocollageneous and lymphoid tissue

**Figure 3:**
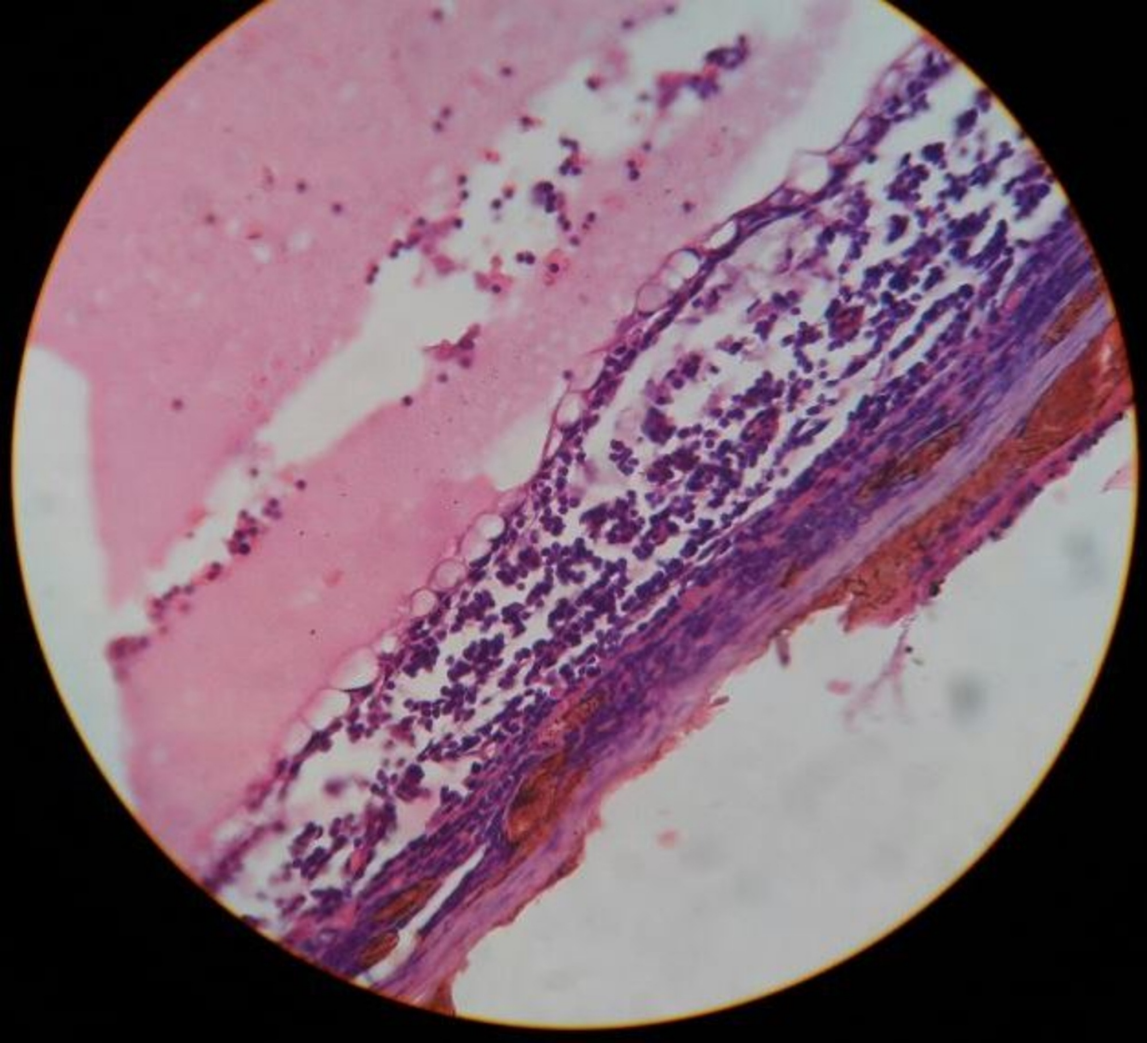
Pathological section shows lining of the cyst that is flattened out The cyst contains proteinaceous material along with scattered lymphocytes and macrophages.

The cyst was excised in toto. The patient recovered well post-operatively. The histopathological examination found the cyst wall had a flattened outlining. The cyst wall had fibrocollagenous and proteinaceous material along with scattered lymphocytes and macrophages (Figures 2-3).

**Figure 4:**
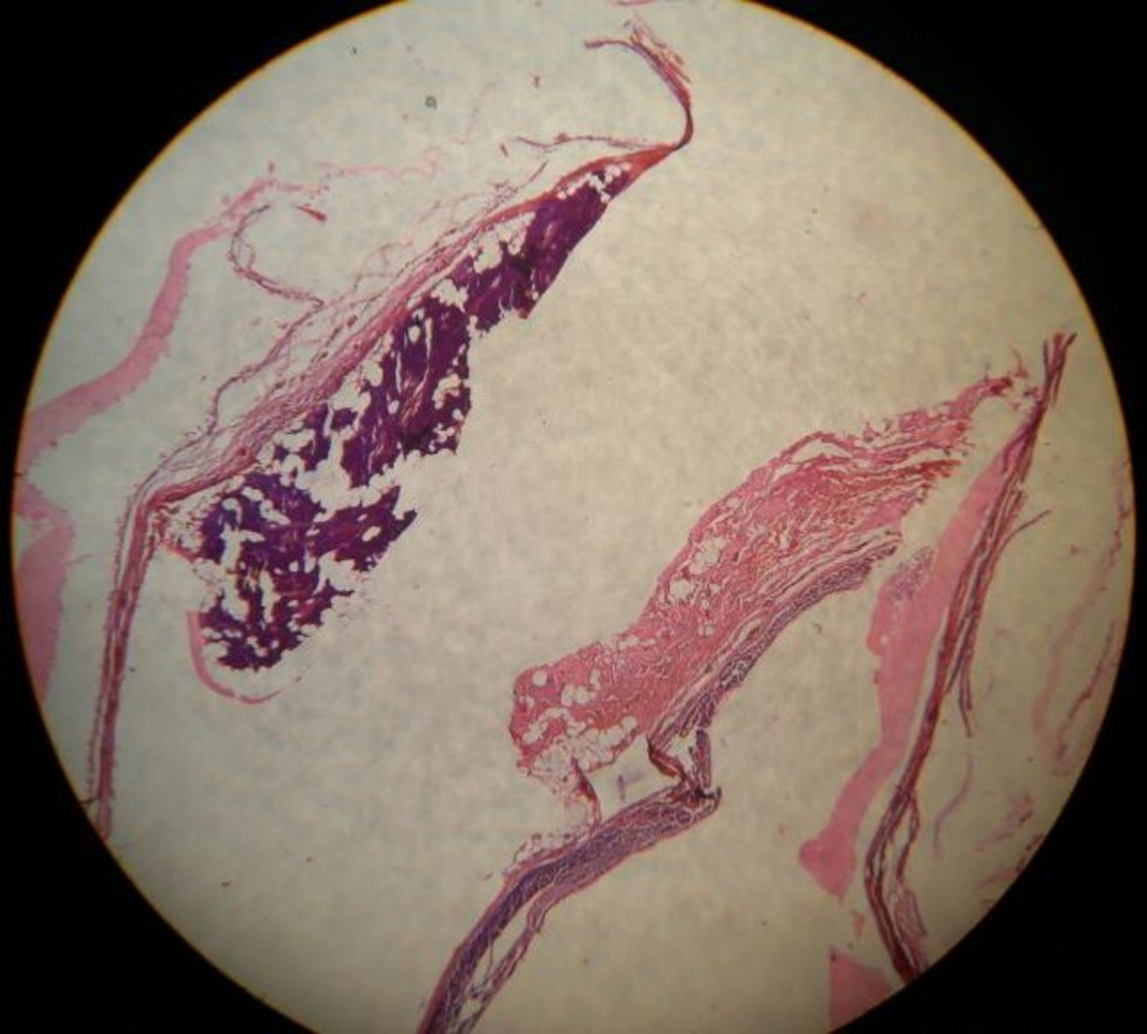
Pathological section shows serous salivary gland tissue along with the adjacent cyst wall

There was serous salivary gland tissue along with some adjacent cyst wall suggestive of lymphangioma of the parotid gland (Figure 4).

The patient was kept under medical surveillance for six months to watch for any local recurrence, but none occurred.

## Discussion

Lymphangiomas are benign developmental abnormalities of the lymphatic system [2]. During the sixth week of embryogenesis, six primitive lymphatic sacs develop. Later, all the lymphatic sacs work together and develop into the mature lymphatic system. Failure of one of the lymph sacs to connect with the rest of the sacs leads to sequestration of lymph and the formation of a cyst. Lymphangiomas are categorized into three types: capillary, cavernous, and cystic. The latter has the potential to infiltrate the surrounding tissue leading to a problematic surgical removal [3].

Lymphangioma occurs in the head and neck region most commonly, accounting for about 90% of cases. Among those found in the head and neck region, the posterior triangle of the neck is frequently involved. Other less commonly reported sites include axilla, shoulder, chest wall, mediastinum, abdominal wall, and thigh. Men and women are equally likely to be affected. Most cases are congenital and 90% present within the first two years of age [4]. It is rare for lymphangiomas to occur in an older population. A history of trauma, infection, iatrogenic injuries, or chronic inflammation may be found in some cases. Clinically, patients typically present with a painless, soft, cystic swelling that enlarges over time. Fluctuation and transillumination of the parotid gland will test positive in cases of lymphangioma.

USG of a lymphangioma will typically show a multicystic, hypoechoic lesion with thin walls [5]. Computed tomography (CT) will reveal a homogeneous cystic lesion with a surrounding smooth, thin wall. CT can also show the involvement of the deep lobe and enhancing pattern of the cyst. For best lymphangioma visualization, magnetic resonance imaging (MRI) is the diagnostic modality of choice. MRI typically displays a thin-walled multicystic lesion hypointense in T1-weighed images and hyperintense in T2-weighed images [6]. Cytologic findings are characterized by hypocellular clear fluid with a number of lymphocytes at various stages, as well as benign parotid epithelium [4].

The cyst may present for a long duration without any symptoms. Complications of the cyst include the risk of infection or hemorrhage leading to the effects of compression on the facial nerve and/or cyst rupture. There are no reported cases of malignancy associated with cystic lymphangioma [3].

Conservative treatment can be safely offered to elderly patients exhibiting no symptoms. Though spontaneous regression of the lymphangioma is a rare phenomenon that has been reported in the literature, typically treatment is necessary for adult patients [7]. Aspiration of the cyst can be done in an emergency to decompress the cyst but recurrence is known to happen. Symptomatic cysts should be excised surgically with complete excision carried out in all cases to reduce the risk of recurrence [8].

Surgical options to consider are enucleation and superficial or total parotidectomy. Surgically, the cyst should be approached with a preauricular incision. Meticulous dissection is needed for complete excision of the cyst. If there is inadequate tumor excision, the rate of recurrence may be high, ranging from 10% to 38% [8]. For patients with a macrocystic lesion unwilling to undergo surgery, sclerosing agents such as OKT-3 and bleomycin may be used with varying results. Intralesional injection with such agents has a role in the medical management of these cases. The use of radiation therapy has no proven role in the management of lymphangioma and adds the risk of malignant transformation [3].

## Conclusions

Lymphangioma is an uncommon congenital lymphatic lesion that uncommonly presents in adults. Most patients are asymptomatic. When symptoms do occur, the lymphangioma requires intervention. USG is recommended for initial non-invasive imaging that leads to a diagnosis. To evaluate the extent of the lesion, MRI is the method of choice. The use of FNAC is often inconclusive, as was seen in our case. Surgical excision of the lesion is often required in the elderly. A definitive diagnosis can be obtained from a final histopathology.
